# Platelet-activating factor and protease-activated receptor 2 cooperate to promote neutrophil recruitment and lung inflammation through nuclear factor-kappa B transactivation

**DOI:** 10.1038/s41598-023-48365-1

**Published:** 2023-12-07

**Authors:** Irismara Sousa Silva, Aline D. Almeida, Antônio C. M. Lima Filho, Weslley Fernandes-Braga, Ayslan Barra, Hortência M. C. Oliveira, Geovanni D. Cassali, Luciano S. A. Capettini, Gustavo B. Menezes, Jacqueline I. Alvarez-Leite, Maria F. Leite, André Klein

**Affiliations:** 1 Laboratory of Inflammation and Proteases, Department of Pharmacology, Institute of Biological Sciences (ICB), Federal University of Minas Gerais (UFMG), Belo Horizonte, Minas Gerais Brazil; 2 Department of Physiology and Biophysics, ICB/UFMG, Belo Horizonte, Minas Gerais Brazil; 3 Laboratory of Atherosclerosis and Nutritional Biochemistry (LABIN-UFMG), Department of Biochemistry and Immunology, ICB/UFMG, Belo Horizonte, Minas Gerais Brazil; 4 Department of Morphology, ICB/UFMG, Belo Horizonte, Minas Gerais Brazil; 5 Department of Pathology, ICB/UFMG, Belo Horizonte, Minas Gerais Brazil; 6 Laboratory of Vascular Biology, Department of Pharmacology, ICB/UFMG, Belo Horizonte, Minas Gerais Brazil

**Keywords:** Asthma, Inflammation, Chronic inflammation, Proteases

## Abstract

Although it is well established that platelet-activated receptor (PAF) and protease-activated receptor 2 (PAR2) play a pivotal role in the pathophysiology of lung and airway inflammatory diseases, a role for a PAR2-PAFR cooperation in lung inflammation has not been investigated. Here, we investigated the role of PAR2 in PAF-induced lung inflammation and neutrophil recruitment in lungs of BALB/c mice. Mice were pretreated with the PAR2 antagonist ENMD1068, PAF receptor (PAFR) antagonist WEB2086, or aprotinin prior to intranasal instillation of carbamyl-PAF (C-PAF) or the PAR2 agonist peptide SLIGRL-NH_2_ (PAR2-AP). Leukocyte infiltration in bronchoalveolar lavage fluid (BALF), C-X-C motif ligand 1 (CXCL)1 and CXCL2 chemokines, myeloperoxidase (MPO), and N-acetyl-glycosaminidase (NAG) levels in BALF, or lung inflammation were evaluated. Intracellular calcium signaling, PAFR/PAR2 physical interaction, and the expression of PAR2 and nuclear factor-kappa B (NF-КB, p65) transcription factor were investigated in RAW 264.7 cells stimulated with C-PAF in the presence or absence of ENMD1068. C-PAF- or PAR2-AP-induced neutrophil recruitment into lungs was inhibited in mice pretreated with ENMD1068 and aprotinin or WEB2086, respectively. PAR2 blockade impaired C-PAF-induced neutrophil rolling and adhesion, lung inflammation, and production of MPO, NAG, CXCL1, and CXCL2 production in lungs of mice. PAFR activation reduced PAR2 expression and physical interaction of PAR2 and PAFR; co-activation is required for PAFR/PAR2 physical interaction. PAR2 blockade impaired C-PAF-induced calcium signal and NF-κB p65 translocation in RAW 264.7 murine macrophages. This study provides the first evidence for a cooperation between PAFR and PAR2 mediating neutrophil recruitment, lung inflammation, and macrophage activation.

## Introduction

Lung inflammation comprises a defense mechanism of the innate and adaptative immune systems to the constant exposure to diverse potentially harmful agents, including pathogens, pollutants, and allergens, aerially dispersed in the environment. Uncontrolled inflammation is the basis of a variety of pulmonary and airway diseases including cystic fibrosis, acute respiratory distress syndrome, chronic obstructive pulmonary disease, and asthma^1^. Therefore, it is assumed that the exacerbated activation of resident and infiltrating leukocytes, which releases bacterial and non-bacterial proteases, proinflammatory cytokines, chemokines and lipidic mediators, promotes an imbalance in inflammatory mechanisms, notably the production of proinflammatory and anti-inflammatory mediators, leading to the lung and airway integrity injury^2^. As occurs in other inflammatory diseases, neutrophil recruitment from the blood vessels to inflamed lung is key in the onset and establishment of lung inflammation. Once in the inflamed tissue, neutrophils play an important role in the progression of tissue damage, contributing to the severity of inflammatory diseases, at least in part through the release of proteases contained in their azurophilic granules, lysosomal enzymes, and reactive oxygen species^2,3^.

Platelet-activating factor (PAF, 1-O-alkyl-2-acetyl-sn-glycero-3-phosphocholine) is a neutrophil chemoattractant lipid mediator implicated in physiology and inflammatory diseases^3^. PAF was first described in the 1970’s^4,5^ as a soluble mediator implicated in anaphylaxis and platelets. Studies have revealed the key roles of PAF in the pathophysiology of inflammatory diseases, such as asthma, allergic diseases, and colitis, as well as in sepsis^6–9^. Pathophysiological levels of PAF in inflammation, are sustained by the synthesis of PAF by resident or effector cells, such as mast cells, basophils, monocytes, eosinophils, epithelial cells, endothelial cells, and neutrophils^3,10^. This involves the activation of phospholipase A_2_ acting on phosphatidylcholine leading to the production of lysophosphatidylcholine, with subsequent addition of acetyl residue mediated by acetyl-CoA lyso-PAF acetyltransferase^11^. PAF acts on the PAF receptor (PAFR), a G protein-coupled receptor (GPCR) expressed on the surface of monocytes, macrophages, neutrophils, eosinophils, and endothelial cells^12^. This receptor is increased in bronchoalveolar lavage fluid (BALF) obtained from asthmatics^8,13^, and their activation triggers the release of cytokines and chemokines, leukocyte recruitment into airway, platelet aggregation, vasodilation, increased vascular permeability, oxidative stress, bronchial hyperactivity, and bronchoconstriction^14,15^. However, interestingly, although PAF plays a pivotal role as a mediator in asthma and other pulmonary diseases, PAFR antagonists have not shown clinical efficacy, suggesting that the activation of additional receptors and mechanisms may be necessary to promote a complete PAF response^7^. Thus, new clinical approaches acting on different targets of inflammatory cascade may be useful for the treatment of lung inflammatory diseases.

Protease-activated receptor 2 (PAR2) and their endogenous activating proteases have emerged as a potential target for the pharmacological control of neutrophil and eosinophil recruitment, or leukocyte activation in several experimental airway inflammation models and in human asthma. PAR2 and their activating proteases mediate the allergen-induced bronchial hyperresponsiveness and activation of inflammatory effector cells^16–23^. This receptor is a member of a family of singular GPCRs (PAR1-4), activated through the proteolytic and irreversible cleavage of a specific fragment of an amino group in the extracellular N-terminal domain of the receptor itself. The cleavage exposes a novel N-terminal peptide sequence that interacts with conserved regions on the receptor's own second extracellular loop, triggering downstream cellular signaling events^24^. The receptor is widely expressed in lungs and upper airway on the surface of vascular smooth muscle cells, airway endothelial and epithelial cells, mast cells (MC), alveolar macrophages, activated neutrophils and eosinophils, and the bronchial epithelium in asthmatics^25–27^. Inhaled allergens and pathogens trigger the release or generation in the airways of pathogen or allergen-derived proteases, trypsin, MC tryptase, neutrophil elastase, cathepsin G, and other PAR2 activating proteases^19–27^. These in turn lead to vasodilation, edema, releasing of proinflammatory cytokines, and leukocyte recruitment^24,27–29^.

PAR2, PAR2-activating proteases, and PAF share the same inflammatory microenvironment and are abundantly present in lung inflammation^17^. Emerging evidence suggests crosstalk between PARs and PAFR driving the inflammatory response through the regulation of the expression of melanoma cell adhesion molecule in the lung^30,31^. Furthermore, patients with difficult to control severe asthma show an enlargement of airway smooth muscle associated with PAR2 overexpression, as well as high levels of PAR2-ligands recovered in BALF, PAR2-knockout mice show lower levels airway hyperresponsiveness and airway inflammation in comparison to wild type mice, and monoclonal anti-PAR2 treatment reduces airway inflammation in an asthma model in mice^27^.

Despite the evidence for a PAR2-PAFR cooperation, a role for this cooperation in lung inflammation has not been investigated. In this study, we investigated possible crosstalk between PAFR and PAR2 mediating neutrophil migration, macrophage activation and lung inflammation in mice.

## Results

### PAR2 participates in PAF-induced neutrophil migration

**Figure 1:**
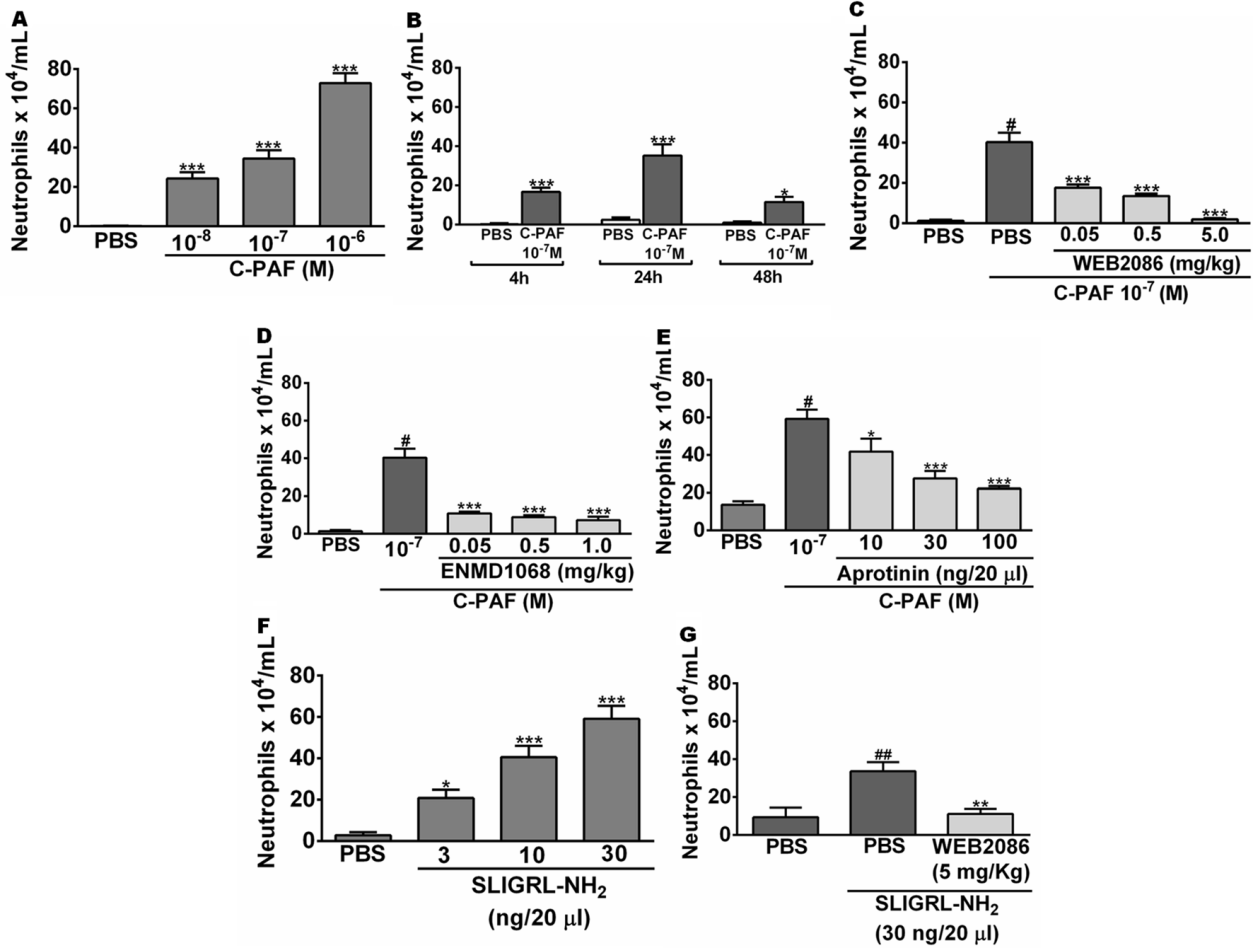
PAR2 blockade and inhibition of proteases impair PAF-induced neutrophil migration in BALB/c mice. (**A**,**B**) Mice were instilled intranasally with PBS or C-PAF. The number of infiltrating neutrophils was counted in BALF 4, 24, or 48 h after challenge. (**C**–**G**) Mice were pretreated with an intraperitoneal injection of WEB 2086 or ENMD1068, or with intranasal instillation of PBS or aprotinin 1 h before intranasal instillation of C-PAF or SLIGRL-NH_2_. The number of infiltrating neutrophils was counted in BALF 24 h after challenge. The results are representative of three independent experiments and are expressed as mean ± SEM from six mice per group. Statistical analysis was assessed by ANOVA followed by Newman-Keuls test, A, B and F: ***p < 0.001 and *p < 0.05 compared to control group (PBS); C, D, E and G: ***p < 0.001, **p < 0.01 and *p < 0.05 compared to C-PAF group; C, D, E and F: ^#^*p* < 0.001 or ^##^*p* < 0.01 compared to control group (PBS).

Our initial experiments were performed to investigate the ability of intranasally instilled PAF to induce neutrophil recruitment into lung. The number of leukocytes recovered in BALF obtained from C-PAF-instilled BALB/c mice was analyzed to assess the importance of PAR2 on PAF-induced neutrophil recruitment. Intranasal instillation of the long-lasting synthetic PAF analog C-PAF increased the number of neutrophils in BALF in all doses utilized (10^–8^–10^–6^ M) compared to PBS-instilled mice group, peaking 24 h after 10^–7^ M C-PAF instillation. Confirming neutrophil recruitment in PAF-instilled mice was due to PAFR activation, pretreatment of mice with the selective PAFR antagonist WEB 2086 prior to C-PAF intranasal instillation abolished neutrophils recovered in BALF 24 h later at all doses tested (Fig. 1A–C). In addition, the number of neutrophils recovered in BALF obtained from C-PAF-instilled mice was strongly impaired in mice pretreated with the selective PAR2 antagonist ENMD1068, as well as by treatment with the strongly competitive and reversible inhibitor of the kallikrein-kinin system and serine proteases aprotinin (10–100 ng/20 µL). The findings suggest that PAR2 is essential in mediating neutrophil recruitment induced by PAF, and that PAR2 endogenous activating proteases released in response to PAF instillation may be responsible for this recruitment (Fig. 1D,E). Interestingly, intranasal instillation of synthetic PAR2 agonist peptide (PAR2 AP) SLIGRL-NH_2_ (3–30 ng/20 µL) selectively increased the number of neutrophils recovered in BALF from C-PAF-instilled mice compared to PBS-instilled mice in all doses utilized. This effect was abolished by pretreatment with the PAFR antagonist WEB 2086 (Fig. 1F,G).

**Figure 2:**
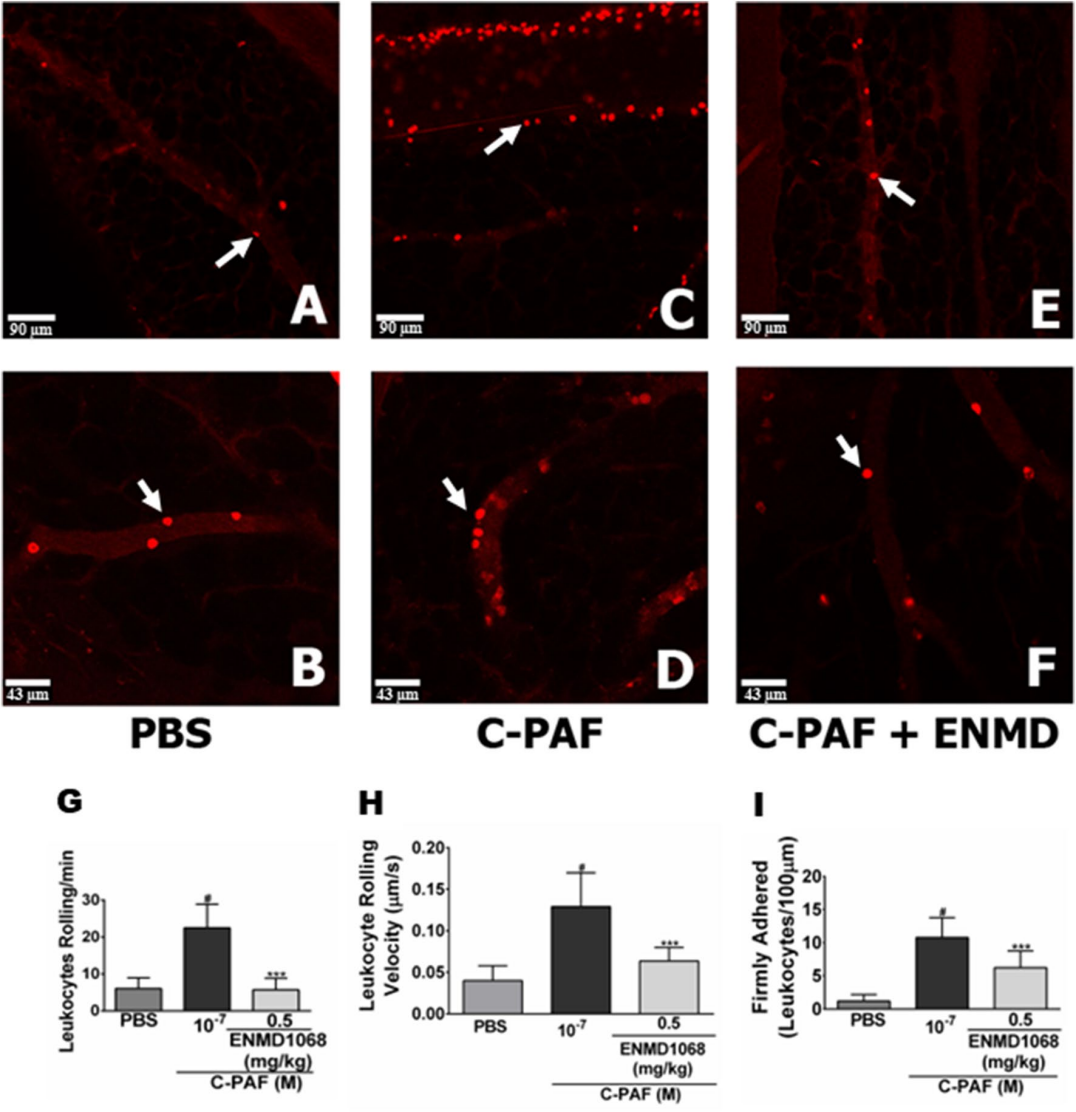
PAR2 blockade impairs rolling and adhesion of leukocytes in the mesentery microcirculation of mice. (**A**–**F**) Representative imaging of mesentery microcirculation by intravital microscopy (neutrophils in red, anti-Ly6G/Ly6C-Gr-1; 0.2 mg/mL, i.v.). Mice were pretreated with ENMD1068 (0.5 mg/kg, i.p.) 1 h before stimulation with C-PAF (10^−7^ M, i.p.). After 4 h, rolling (**G**), velocity (**H**), and adhesion (**I**) of leukocytes (white arrows) were evaluated in the venules of mesentery microcirculation. The fluorescence intensity was monitored for 10 min using an objective Plan Apo 20 × calibrated by each venule. The results are representative of three independent experiments with similar results and are expressed as mean ± SEM from six mice per group. Statistical analysis was assessed by ANOVA followed by Newman-Keuls test, (**G**–**I**) ***p < 0.001 compared to C-PAF group and ^#^*p* < 0.001 compared to control group (PBS). Scale bar: 90 μm or 43 μm.

To assess and separately study the effects of PAR2 on leukocytes in microcirculation, intravital and confocal microscopy studies in mesentery microcirculation were performed using a binding antibody (anti-Ly6G/Gr-1) that recognize the surface molecule Gr-1 mainly expressed in neutrophils/monocytes. Analysis of the mesentery microcirculation revealed very few leukocytes surrounding the blood vessel in PBS-injected mice (Fig. 2A,B, and Supplementary Video 1), while the number of leukocytes surrounding the vessels increased after C-PAF instillation (10^−7^ M, Fig. 2C,D, and Supplementary Video 2). By contrast, the number of leukocytes in the microcirculation was inhibited after treatment mice with the PAR2 antagonist ENMD 1068 (Fig. 2E,F, and Supplementary Video 3). The number of adherent rolling, velocity and adherent leukocytes in the mesentery microcirculation was reduced following PAR2 antagonist treatment in C-PAF-treated mice (Fig. 2G–I).

### PAR2 blockade impairs C-PAF-induced lung inflammation and production of neutrophil chemokines CXCL1 and CXCL2 in BALB/c mice

**Figure 3:**
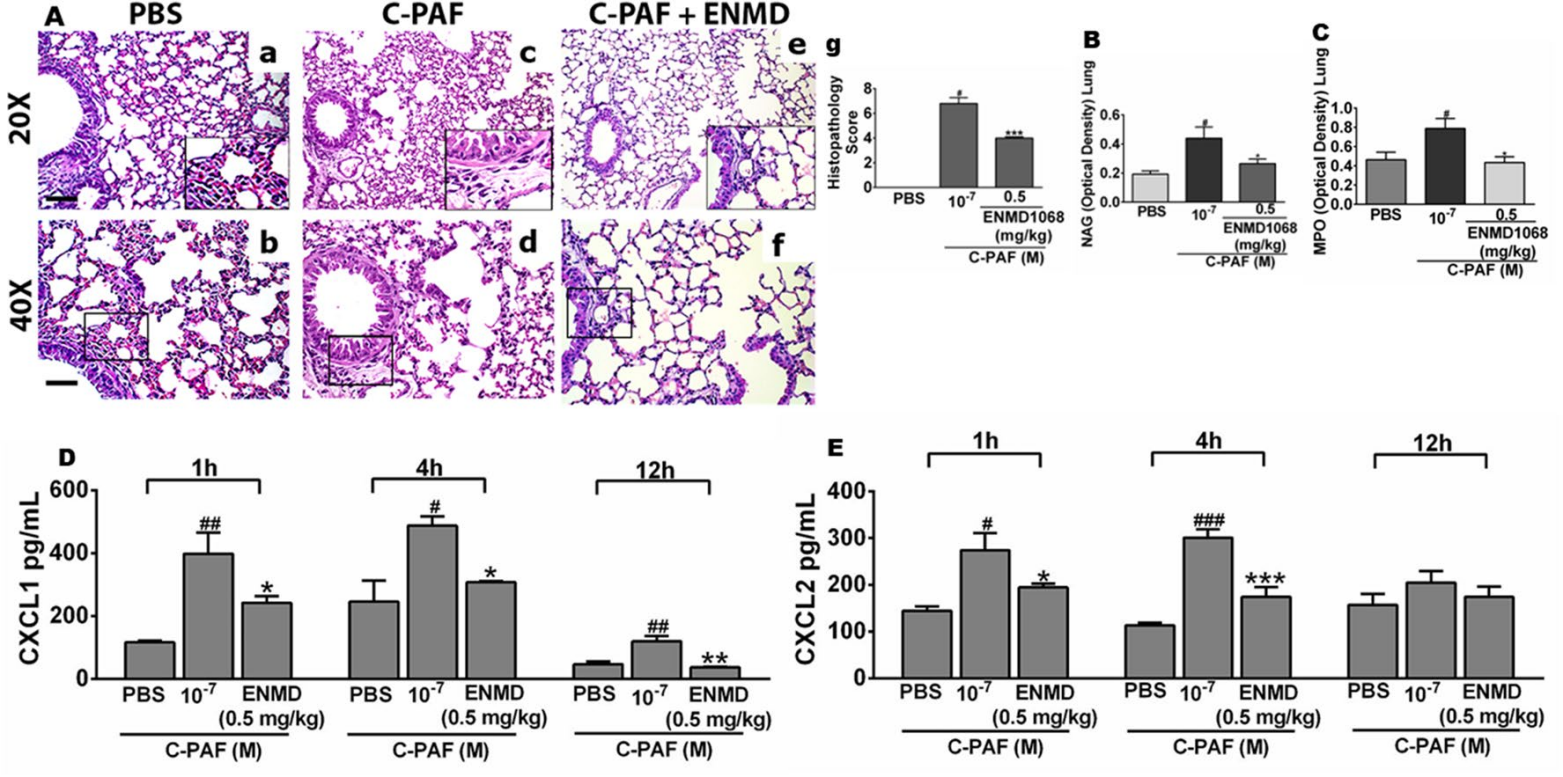
PAR2 blockade reduces C-PAF-induced lung inflammation and production of chemokines in BALB/c mice. (**A**) Mice were pretreated with ENMD1068 (0.5 mg/kg, i.p.). One hour later the mice were stimulated with C-PAF (10^–7^ M; i.n.). After 24 h, the lungs were collected and stained with H&E (20 × scale of 100 μM; 40 × scale of 50 μM). Panel a-b: PBS group, which exhibits normal histological features and no evidence of lung inflammation; Panel c-d: C-PAF group showing perivascular and peribronchiolar inflammation; Panel e–g: ENMD1068 pretreated group, shows cellular infiltration reduced as assessed by the histopathology score. (**B**–**E**) mice were pretreated with ENMD1068 (0.5 mg/kg, i.p.) 1 h prior to administration of C-PAF (10^–7^ M; i.n.). NAG and MPO levels were evaluated 24 h later and CXCL1 and CXCL2 levels were assessed 1, 4, and 12 h later. The results are representative of three independent experiments and expressed as mean ± SEM from six mice per group. Statistical analysis was assessed by ANOVA followed by Newman-Keuls test, Panel g: ***p < 0.001 compared to C-PAF group and ^#^*p* < 0.001 compared to control group (PBS); (B–E) ***p < 0.001, **p < 0.01 and *p < 0.05 compared to C-PAF group and ^#^*p* < 0.001, ^##^*p* < 0.01 and ^###^*p* < 0.05 compared to control group (PBS).

The impact of PAR2 blockade on lung inflammation was investigated through the lung histopathological analysis and detection of MPO and NAG in lung tissue obtained from PAF-instilled mice. There was no inflammation in lungs obtained from PBS-instilled mice (Fig. 3A panels a, b). Lungs obtained from C-PAF-instilled mice displayed perivascular and peribronchiolar inflammation with leukocyte infiltration after 24 h (Fig. 3A panels c, d). Pretreatment of mice with the PAR2 antagonist ENMD-1068 markedly reduced cellular infiltration in the perivascular region (Fig. 3A panels e, f), impaired lung inflammation as assessed by the histopathology score (Fig. 3A panel g). Moreover, PAR2 blockade with ENMD1068 reduced levels of both enzymes compared to C-PAF-instilled mice (Fig. 3B and C). Supporting these results, ENMD1068 treatment decreased the levels of the neutrophil chemokines CXCL1 and CXCL2 measured in BALF 1 h or 4 h after intranasal instillation of C-PAF compared to C-PAF treated mice. Although low levels of CXXL1 were persistent in ENMD1068-treated mice 12 h after C-PAF instillation, CXCL2 returns to basal levels 12 h after C-PAF instillation (Fig. 3D and E). These findings suggest PAR2 activation is required for neutrophil recruitment into lungs in PAF-instilled mice, at least partially due to the release of CXCL1 and CXCL2. Other chemotactic mediators may be responsible for the sustained leukocyte recruitment 24 h after PAF instillation.

### Co-activation of PAFR and PAR2 is required for PAFR/PAR2 physical interaction and PAR2 blockade impairs PAF-induced Ca^2+^ signal in RAW 264.7 murine macrophages

**Figure 4:**
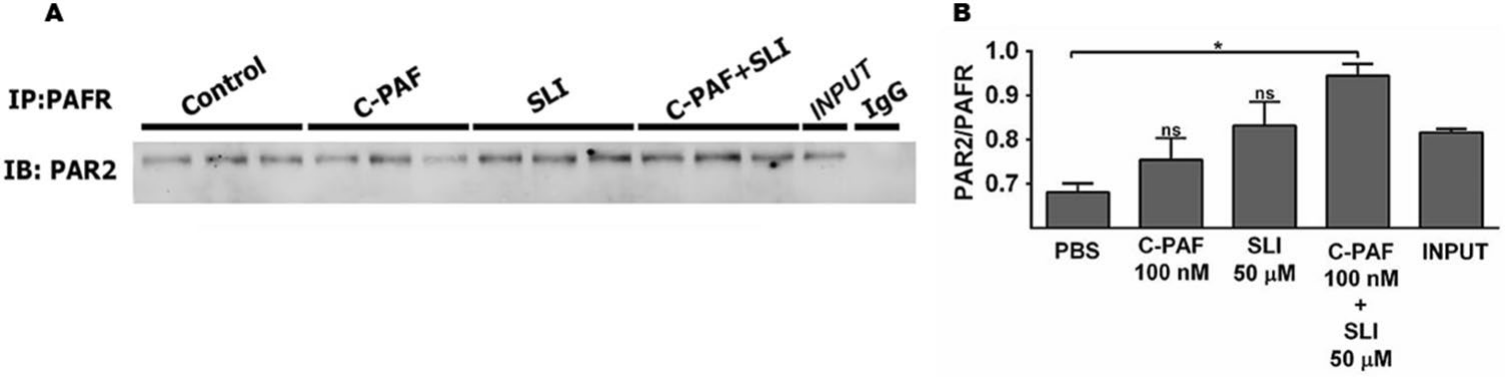
PAFR/PAR2 physical interaction in RAW 264.7 murine macrophages. Cells were stimulated with C-PAF (100 nM), SLIGRL-NH_2_ (50 μM), or simultaneous co-stimulation (C-PAF 100 nM + SLIGRL-NH_2_ 50 μM) for 20 min before addition of RIPA. Cell lysates were subjected to immunoprecipitation (**A**) and west immunoblotting (**B**) using antibodies to PAFR and PAR2. Representative image of the interaction and quantification of PAR2 and PAFR proteins immunoprecipitated. The experiments were performed in duplicate and one membrane was developed with anti-PAR2 and the other with anti-PAFR for normalization control. In data shown, each band in representative membrane image of western blotting correspond of three separate experiments realized before gel electrophoresis running. Values are expressed as mean ± SEM. Statistical analysis was assessed by ANOVA followed by Newman-Keuls test, B: *p < 0.05 compared to control group (PBS).

**Figure 5:**
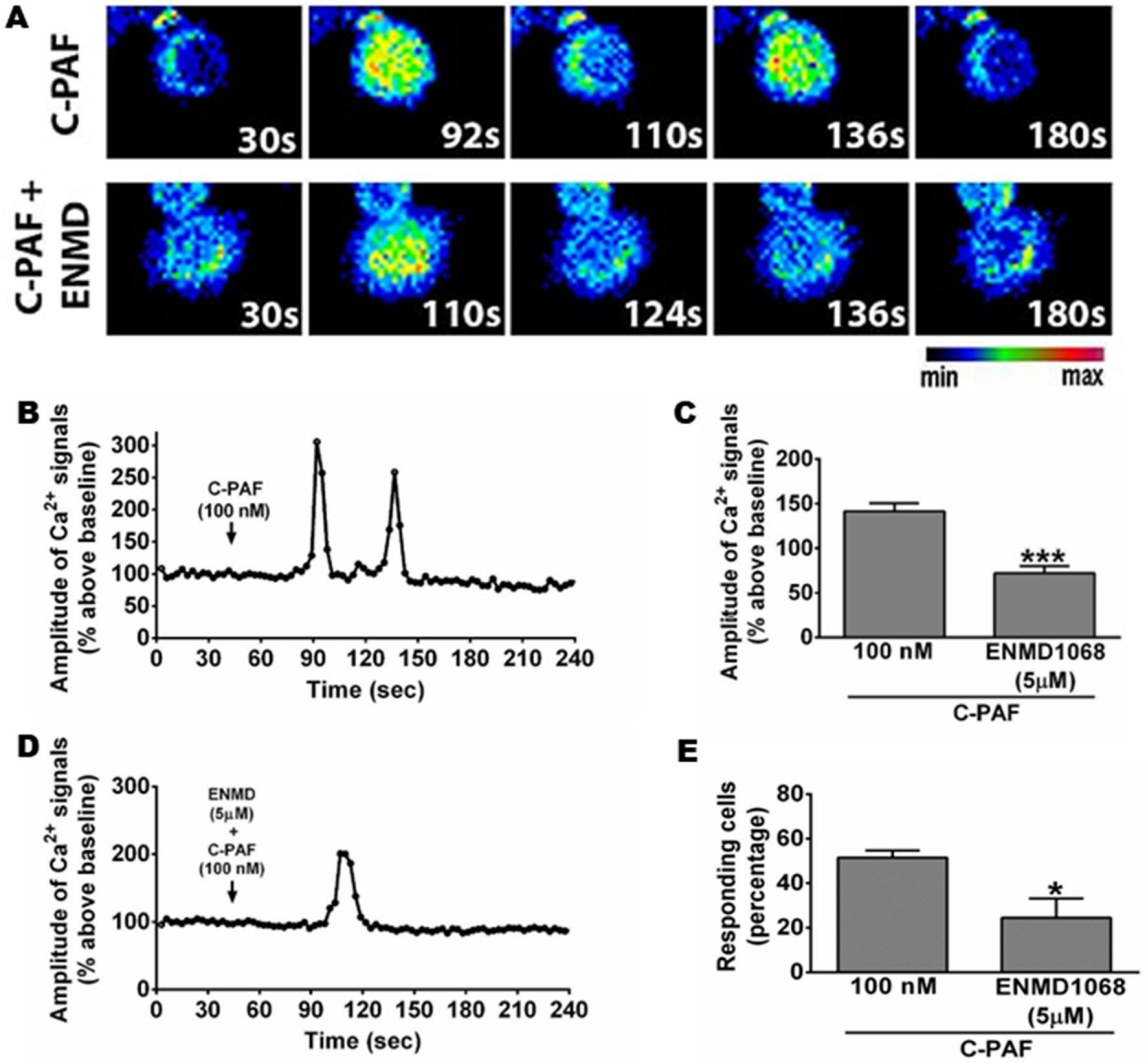
PAR2 blockade reduced C-PAF-induced intracellular Ca^2+^ signals in RAW 264 cells. Cells were pretreated with ENMD1068 (5 μM) 1 h before C-PAF (100 nM) stimulation. (**A**–**C**) Representative cells for Ca^2+^ signal amplitude as a function of time. (**D**) Ca^2+^ signaling amplitude (% above basal) and (**E**) percentage of responsive cells (% above basal). Bars represent mean ± SEM (n = 3 preparations with 30 cells per group). Statistical analysis was by ANOVA followed by Newman-Keuls test, (**C**) and (**E**) ***p < 0.001 and *p < 0.05 compared to C-PAF group.

Our next analyses were performed to evaluate whether PAR2 and PAFR could physically interact through co-immunoprecipitation assay in RAW 264.7 macrophages. Basal coprecipitation between PAFR and PAR2 was evident in resting control macrophages. The level was not increased after PAFR activation with C-PAF alone or PAR2 activation with PAR2 AP alone. Interestingly, PAR2-PAFR immunoprecipitation increased after co-stimulation of these receptors with their respective agonists (Fig. 4A,B). PAFR activation promotes intracellular Ca^2+^ mobilization, through Gαq subunits following activation of G protein complex, triggering cytoskeletal events supporting cell motility and leukocyte changes in shape. Thus, the next series of experiments were designed to evaluate whether PAR2 blockade could influence PAF-induced intracellular Ca^2+^ mobilization. As expected, C-PAF markedly increased the intracellular Ca^2+^ mobilization in RAW 264.7 cells signal, and importantly, implicated PAR2 in the PAF response. The findings support the cooperation between PAR2 and PAFR mediating Ca^2+^ signaling events. PAR2 blockade with ENMD1068 significantly inhibited both the amplitude of the fluorescent signal compared to that of PAF-stimulated cells and the percentage of responding cells compared with that of the C-PAF group (Fig. 5).

### PAFR activation decreases PAR2 expression and PAR2 blockade impairs PAF-induced NF-κB p65 translocation in RAW 264.7 murine macrophages

**Figure 6:**
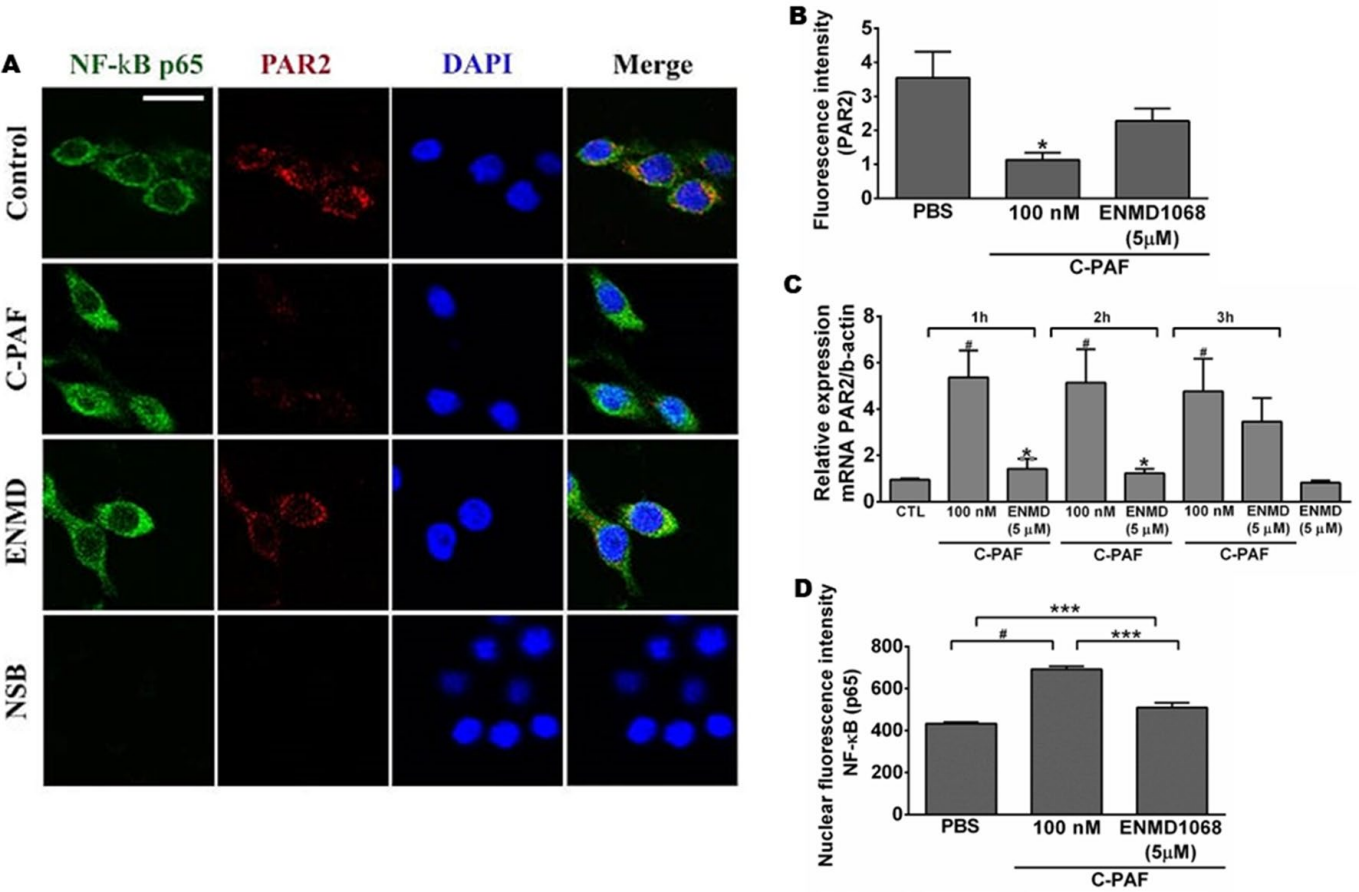
Expression of PAR2 and NF-КB (p65) transcription factor in RAW 264.7 cells. Cells were pretreated with ENMD1068 (5 μM) 1 h prior to C-PAF (100 nM) stimulation, fluorescence intensity was evaluated 4 h later. (**A**) Representative images of PAR2 (red), NF-kB (p65, green) and DAPI (nucleus, blue) expression. (**B**) PAR2 fluorescence intensity. (**C**) Nuclear fluorescence intensity for NF-κB (p65). (**D**) Cells were pretreated with ENMD1068 (5 μM) 1 h prior C-PAF (100 nM) stimulation. PAR2 mRNA was analyzed 1, 2, and 3 h later by qPCR. (**B**,**C**) Bars represent mean ± SEM (n = 3 preparations with 30 cells per group). Statistical analysis was by ANOVA followed by Newman-Keuls test, (**B**) and (**D**) ***p < 0.001 and *p < 0.05 compared to control group (PBS); (**C**) and (**D**) ***p < 0.001 and *p < 0.05 compared to C-PAF group and ^#^*p* < 0.001 compared to control group (PBS).

Having demonstrated cooperation between PAFR and PAR2, we investigated whether PAFR activation could mediate PAR2 expression in RAW 264.7 murine macrophages. PAR2 was constitutively expressed in RAW 264.7 murine macrophages, and PAR2 blockade did not alter PAR2 expression in C-PAF-stimulated cells or non-stimulated cells (Fig. 6). Surprisingly, PAR2 expression was impaired in C-PAF-stimulated cells compared to non-stimulated cells (Fig. 6A and B). To understand the underlying mechanisms involved in this response, the expression of PAR2 mRNA in RAW 264.7 macrophages was investigated 1, 2, or 3 h after C-PAF incubation. C-PAF increased PAR2 mRNA in all analyzed time points compared to non-stimulated cells. Co-incubation of cells with PAR2 antagonist reduced PAR2 mRNA expression (Fig. 6C). Because NF-κB is an important positive regulator in the synthesis of numerous proinflammatory mediators such as cytokines, chemokines and antimicrobial peptides^32^ we investigated the impact of PAR2 blockade on C-PAF-induced NF-κB (p65) translocation. As expected, C-PAF treatment increased the NF-κB (p65) transcription factor nuclear fluorescence compared to control cells. However, PAR2 blockade with PAR2 antagonist ENMD1068 reduced NF-κB (p65) nuclear fluorescence (Fig. 6A and D).

## Discussion

It is well established that PAF plays a pivotal role in the pathophysiology of allergic or non-allergic-induced lung and airway inflammatory diseases, mediating eosinophil and neutrophil recruitment, airway hyperresponsiveness, mucus hypersecretion, gas exchange impairment, and leukocyte activation. These activities contribute to the establishment and progression of inflammatory airway diseases^14,33^. However, the pharmacological blockade of PAFR exhibit modest beneficial effects, if any, in clinical trials of PAFR antagonists, limiting their therapeutic use in humans^7^. PAR2 blockade may be a new approach in the treatment of lung diseases by reduce proinflammatory signaling including leukocyte activation and hyperresponsiveness^18,19,22^. Because PAR2 and PAFR are expressed on the surface of cells committed to lung inflammation sharing the same inflammatory microenvironment, and PAR2 activating proteases are abundantly released in PAF-induced inflammation^1,19,34^, the present study was performed to investigate a possible network cooperativity between PAR2 and PAFR mediating neutrophil recruitment and lung inflammation.

Initially, we investigated the ability of C-PAF, a long-lasting PAF analog, and PAR2-AP SLIGRL-NH_2_ to induce neutrophil recruitment into lungs of mice, the importance of endogenous proteases in mediating C-PAF-induced neutrophil recruitment, and the impact of PAR2 pharmacological blockade on these effects. Neutrophil recruitment from blood vessels into inflamed tissue plays a pivotal role in the pathophysiology of inflammatory lung diseases. This recruitment is triggered by tissue releasing of proinflammatory and chemo attractant molecules including CXCL1/KC, functional mouse homolog of human CXCL8 produced by activated human monocytes, neutrophils, nasal and bronchial epithelium, bronchoalveolar macrophages, and CXCL2/MIP2-alpha functional mouse homolog of human CXCL8 produced by monocytes and macrophages^35–38^. Once at the inflamed tissue, neutrophils contribute to the progression of tissue damage and severity of inflammatory diseases, at least in part through the release of PAR2-activating proteases contained in their azurophilic granules as neutrophil elastase, cathepsin G and proteinase-33^34,39^, lysosomal enzymes and reactive oxygen species^40,41^. Thus, understanding the mechanisms underlying neutrophil recruitment in vivo may aid the development of novel pharmacological strategies for the treatment of lung inflammatory disorders. Herein, the intranasal instillation of C-PAF or PAR2 AP increased the number of neutrophils present in BALF. The effect was abolished in mice treated with PAFR antagonist WEB 2086, and the pharmacological blockade of PAR2 as well as protease inhibitor aprotinin inhibited PAF-induced neutrophil recruitment. These findings suggest that C-PAF-induced neutrophil recruitment is dependent on PAFR and PAR2 activation by proteases, which are inhibited by aprotinin. In addition, PAR2 blockade markedly reduced histopathology score of inflammation and the activity of both macrophages and infiltrating neutrophils in lungs of C-PAF-instilled mice. Our results are consistent with those of a previous study, in which PAR2 antibody reduced allergen-induced airway inflammation and leukocyte migration into lungs^22^. Surprisingly, PAR2-AP-induced neutrophil recruitment was abolished by pharmacological blockade of PAFR. Thus, we hypothesize that in lung inflammation the activation of PAR2 by endogenous proteases released following cellular damage may exert a proinflammatory regulation on neutrophils, acting in cooperation with PAFR to promote the neutrophil recruitment into the lung.

Consistent with our findings in C-PAF-induced neutrophil recruitment and lung inflammation, and the anti-inflammatory effects evoked by PAR2 blockade, we demonstrate that PAR2 blockade impairs the rolling and adherence of monocytes and neutrophils in the mesentery microcirculation of C-PAF treated mice, thereby impairing the ability of leukocytes to migrate from blood vessels into tissues. It has been demonstrated that the pharmacological blockade or the genetic depletion of PAFR impairs rolling and adhesion of leukocytes^42^, and PAF elicits the production of the neutrophil chemokines CXCL1 and CXCL2^43,44^. However, to the best of our knowledge, we are the first to demonstrate that PAR2 blockade impairs C-PAF-induced neutrophil adhesion and rolling, CXCL1 and CXCL2 lung production, suggesting that PAR2 blockade may be useful in inflammatory diseases where PAF plays a central role on the development and progression of the inflammatory disease through the impairment of early events of lung diseases, such as neutrophil migration.

The physical interaction between PAR2 and other receptors committed to inflammatory/innate response has been previously reported. For instance, PAR2 physically interacts with gram-negative bacteria-induced Toll-like receptor 4 (TLR4) and cooperates downstream of proinflammatory signaling through the activation of the nuclear transcriptional factor NF-κB^28^. In this study, we addressed a possible PAR2/PAFR physical interaction following their activation in a co-immunoprecipitation assay. Co-activation of PAR2 and PAFR led to their physical interaction in RAW 264.7 murine macrophages. This suggests that PAR2/PAFR interaction may be necessary to enhance proinflammatory signaling. Our results are consistent with previous findings that the activation of the thrombin receptor PAR1 induces the expression of PAFR in melanoma cells, with PAR1 and PAFR cooperating to promote transendothelial migration of melanoma cells with the accumulation of melanoma cells in the lung^30,31^. Because PAFR activation induces intracellular Ca^2+^ mobilization through activation of G protein by Gαq subunits, which in turn triggers the intracellular inositol triphosphate production^45,46^ we investigated whether PAR2 blockade could promote a shift in C-PAF-induced intracellular Ca^2+^ mobilization in RAW 264.7 murine macrophages. Interestingly, pharmacological PAR2 blockade reduced intracellular Ca^2+^ signal amplitude and responsiveness of macrophages to C-PAF stimulation, suggesting a cooperation between PAR2 and PAFR mediating intracellular signaling via intracellular Ca^2+^ mobilization. We hypothesize that in an inflammatory microenvironment rich in PAF and PAR2-activating proteases released following cellular damage by competent immune/innate cells, such as macrophages, PAR2 and PAFR co-activation drives a physical PAR2/PAFR interaction, which in turn amplifies the inflammatory response by increasing neutrophil recruitment.

PAFR activation leads to the expression of the transcription factor NF-κB (p65)^47–49^, and activation of the NF-κB subunits is critical for proinflammatory gene expression^50^. We observed that the pharmacological blockade of PAR2 impaired the activation of NF-κB (p65) in C-PAF-stimulated RAW 264.7 murine macrophages. Thus, we propose that a PAR2 activation may be at least partially required to promote NF-κB activation in response to C-PAF, triggering the PAF-mediated proinflammatory downstream intracellular signaling. These results strongly indicate a pivotal role for PAR2 activation in C-PAF-induced NF-κB (p65) expression. To clarify the underlying mechanisms of this PAR2/PAFR cooperation, we investigated whether C-PAF may increase PAR2 expression in C-PAF-stimulated RAW 264.7 murine macrophages. Interestingly, although PAR2 mRNA expression increases 3 h after incubation of cells with C-PAF, and PAR2 protein expression was reduced 4 h after C-PAF addition to the culture, pre-incubation of cells with PAR2 antagonist did not change the C-PAF-induced PAR2 expression. Collectively, these data indicate that PAR2 protein expression reduction was not due to PAR2 mRNA expression reduction. Mechanisms of irreversible activation PAR2 that involve receptor endocytosis, recycling or degradation through a β-arrestin-dependent classic mechanism^51,52^, may be involved in this effect. Based on these results, we hypothesized that 4h after macrophage exposition to C-PAF, PAR-2-activating proteases trigger an irreversible PAR2 activation which in turn leads to β-arrestin-mediated endocytosis to downstream recycling or degradation.

In conclusion, this work demonstrates for the first-time the cooperation between PAFR and PAR2 that mediates neutrophil recruitment and lung inflammation, and a PAR2/PAFR interactive network mediating intracellular signaling events as Ca^2+^ mobilization and NF-κB (p65) activation in murine macrophages. Further studies are necessary to clarify the complexity of this interaction as well as its pathophysiological significance to the inflammatory lung diseases. However, the present study provides new insight to understand the failure of PAFR antagonists in the clinical use in asthma in humans, since a combined therapy based on targeting PAFR and PAR2 would be more efficient as a new pharmacological approach regarding the treatment of inflammatory lung diseases.

## Methods

### Mice

All animal protocols were approved by Animal Use Ethics Committee of Federal University of Minas Gerais—UFMG (CEUA, 348/14). Male BALB/c mice (25–30 g, 8–10 weeks old, randomly assigned to experimental groups of equal size) obtained from the Bioterism Center of the Institute of Biological Sciences of UFMG (CEBIO-ICB/UFMG), housed five per cage (194 mm × 181 mm × 398 mm) at a temperature of 25 ± 2 °C under a 12/12-h light/dark cycle with food and water ad libitum. Throughout the experiments, the mice were managed in accordance with the principles and guidelines for the care of laboratory animals.

### Drugs and reagents

Mouse CXCL1 (Cat# DY453-05) and CXCL2 (Cat# MM200) ELISA kit were obtained from R&D Systems (Minneapolis, MN, USA, RRID:SCR_006140). The Carbamyl-PAF (1-O-palmitol2-(N-methylcarbamyl)-sn-glycero-3-phosphocholine, Cat# BML-L120-0005), WEB2086 (Cat# BML-L137-0005) and PAR2 antagonist ENMD-1068 (Cat# BML-N110-0005) were purchased from Enzo Life Sciences (Ann Arbor, MI, USA; RRID:SCR_003900). The PAR2-agonist peptide (AP) SLIGRL-NH_2_ (Cat# 1468) was obtained from Tocris Bioscience (Bristol, UK; RRID:SCR_003689). Aprotinin (Cat# 616370) and PureProteome™ Protein A/G Mix Magnetic Beads (Cat# LSKMAGAG02) were obtained from Merck (New Jersey, USA; RRID:SCR_001287). PE-conjugated anti-Ly6G/Ly6C (Cat# 12-5931-85, RRID: AB_466047) was obtained from eBioscence/ Thermo Fisher Scientific (Waltham, MA, USA). Anti-PAR2 polyclonal antibody (Cat# ab124227) and Anti-PAFR polyclonal antibody (Cat# ab104162, RRID) were obtained from Abcam (Cambridge, UK). Anti-NF-kB (p65) (Cat# sc-8008; RRID:AB_628017) and normal rabbit IgG (Cat# sc-2027; RRID:AB_737197) were purchased from Santa Cruz Biotechnology (Dallas, TX, USA; RRID:SCR_008987). Fluo-4/AM (Cat# F1420) and DAPI (Cat# D21490) were obtained from Invitrogen (Carlsbad, CA, USA; RRID:SCR_008410). All drugs were dissolved in phosphate-buffered saline (PBS, pH 7.4). Details of other materials and suppliers are provided in the specific sections.

### Cell culture

The RAW 264.7 (ATCC Cat# TIB-71, RRID: CVCL_0493, passages 2–10) a murine macrophage cell line was obtained from the American Type Culture Collection (ATCC TIB-71, Rockville, USA) and was grown in Dulbecco's modified Eagle's médium—DMEM, (Gibco Thermo Fisher Scientific, Gaithersburg, MD, USA; Cat# C11995500CP), supplemented with 10% fetal bovine serum and 1% penicillin/streptomycin (Gibco Thermo Fisher Scientific, Gaithersburg, MD, USA; Cat# A3382001). The cells were maintained at 37°C in a humid atmosphere with 5% CO_2_ and were passaged every 2 to 3 days, upon reaching 80–90% confluency. All experiments were performed using cells with passages between 2 and 10, after determining the viable cell concentration using a hemocytometer and trypan blue dye.

### Leukocyte infiltration in mice BALF

BALF was obtained in rabbits as first described by Myrvik et al.^53^, and, adapted for mice. Briefly, mice were intraperitoneally (i.p.) pretreated with WEB2086 (0.05–5.0 mg/kg), ENMD1068 (0.05–1.0 mg/kg), or with intranasal (i.n.) instillation of aprotinin (10–100 ng/20 µL) 1 h prior to i.p. administration of anesthetic (ketamine, 75 mg/kg and xylazine, 10 mg/kg) and immediate challenge with i.n. instillation of C-PAF (10^–8^–10^–6^ M/20 µL), SLIGRL-NH_2_ (3–30 ng/20 µL) or PBS at a volume of 20 μL. ENMD1068, aprotinin, C-PAF and PAR2-AP SLIGRL-NH_2_ doses were based on previous studies^10,20,23,54–58^. Twenty four hours later, the mice were terminally anesthesized by i.p. administration of a ketamine/xylazine solution (20 μL/kg). A catheter was inserted into the trachea, and a total of 2 mL of PBS was used for perfusion and aspiration of BALF in each mouse. The recovered BALF was centrifuged at 100 g for 5 min at 4°C. The pellet was used to determine the total cell counts by using a modified Neubauer chamber and Turk’s stain. Differential cell counts were performed on cytospin preparations stained with May-Grunwald and Giemsa using standard morphological criteria to identify cell types. The results are presented as the number of cells per mL.

### Histological evaluation

Mice were pretreated with ENMD1068 (0.5 mg/kg, i.p.) 1 h before challenge with C-PAF (10^–7^ M/20 µL, i.n.). The lungs were removed 24 h after treatments, fixed in a 10% neutral solution of formalin, dehydrated gradually in ethanol, embedded in paraffin, sectioned (3 μm thick), stained with hematoxylin and eosin (H&E), and examined by light microscopy by a pathologist blinded to the experiment. Inflammation scoring (Table 1) was performed by two different and independent investigators, from qualitative analysis of H&E slides based on a previous study^59^ to evaluate airway vascular and parenchymal inflammation and neutrophilic and eosinophilic infiltration (0, absent; 1, slight; 2, moderate; 3, marked; and 4, severe).

**Table 1:** Histopathological scoring system for mouse lungs (maximum of 15) to evaluate airways, vascular and parenchymal inflammation, and neutrophil infiltrating.

Points	Score 1	Score 2	Score 3	Score 4	Total
	Airway inflammation	Vascular inflammation	Parenchymal inflammation	Neutrophil infiltrating	
0 Absent	Lack of inflammatory cells around airways	Lack of inflammatory cells around vessels	< 1% affected	No neutrophils around airways	
1 Slight	Some airways have small numbers of cells	Some vessels have small numbers of cells	1–30% affected	Few neutrophils around airways	
2 Moderate	Some airways have significant inflammation	Some vessels have significant inflammation	30–60% affected	Some neutrophils in airways	
3 Marked	Majority of airways have some inflammation	Majority of vessels have some inflammation	> 60% affected	Many neutrophils in airways	
4 Severe	Majority of airways are significantly inflamed	Majority of vessels are significantly inflamed	–	Airways are significantly inflamed	
Total	4	4	3	4	15

Inflammation scoring was performed by two different and independent investigators, from qualitative analysis of H&E slides, to evaluate airway vascular and parenchymal inflammation and neutrophilic and eosinophilic infiltration (0, absent; 1, slight; 2, moderate; 3, marked; and 4, severe).

### Myeloperoxidase (MPO) and N-acetyl-glucosaminidase (NAG) measurement

Myeloperoxidase and NAG are constituents of the azurophilic granules of neutrophils or macrophages that oxidizes chloride ions to the potent bactericidal oxidant hypochlorous acid (HOCl) acting as a host defense mechanism by efficiently mediating microbial killing and have been considered as markers of neutrophil or macrophage activity in inflamed tissue^60^. MPO activity reaction was started at 37 °C for 5 min in a 96-well microplate by adding the supernatant and 25 µL of 3,3´-5,5´-tetramethylbenzidine (TMB, Cat# 860336; Sigma-Aldrich, St. Louis, MO, USA); dissolved in dimethyl sulfoxide (Merck, New Jersey, NJ, USA; Cat# 317275) in a final concentration of 1.6 mM, 100 µL hydrogen peroxide (H_2_O_2_), dissolved in phosphate buffer (0.05 M Na_3_PO_4_, 0.5% hexadecyltrimethylammonium bromide, pH 5.4) in a final concentration of 0.003% v/v and 25 µL supernatant from tissue sample processing. After that, H_2_O_2_ was added and the mixture incubated at 37°C for 5 min. The reaction was stopped by adding 100 µL of 4 M H_2_SO_4_ and was quantified at 450 nm using a SpectraMax Plus 384 spectrophotometer (Molecular Devices, RRID:SCR_018598). The NAG activity reaction was started at 37°C for 10 min in a 96-well microplate by the addition of 100 µL *p*-nitrophenyl-N-acetyl-β-D-glucosaminide (Sigma-Aldrich, St. Louis, MO, USA; Cat# 487052), dissolved in citrate/phosphate buffer (0.1 M citric acid, 0.1 M Na_2_HPO_4_, pH 4.5) in a final concentration of 2.24 mM in 100 µL of supernatant from tissue sample processing, dissolved in citrate/phosphate buffer at appropriate dilutions. The reaction was terminated by the addition of 100 µL 0.2 M glycine buffer (pH 10.6) and was quantified at 405 nm in a spectrophotometer (RRID:SCR_018598).

### Intravital microscopy of leukocytes to assess rolling and adhesion of neutrophils to the mesentery

To study the effects of PAR2 blockade on leukocyte rolling, rolling velocity, and adhesion to mesenteric microcirculation induced by C-PAF, we performed intravital studies in a mouse mesenteric microcirculation, as described previously^61^. This established model in intravital studies allows the visualization and quantification in real-time of rolling leukocytes and adherent leukocytes to the vascular endothelium. Mice were pretreated with PAR2 antagonist ENMD1068 (0.5 mg/kg, i. p.) 1 h before the intranasal instillation of C-PAF (10^–7^ M). Four hours after C-PAF instillation, mice were anesthetized by i.p. injection of a mixture of 10 mg/kg xylazine and 75 mg/kg ketamine hydrochloride. The mesenteric tissue was carefully dissected and exteriorized by midline laparotomy for intravital microscopy studies. After that, mice were maintained in a stable position on an acrylic custom-made mouse stage. The tissue to be transilluminated was positioned on the stage of an Eclipse Ti microscope (Nikon, RRID:SCR_021242) with an A1R confocal (RRID:SCR_020317) head equipped with four different lasers (excitation at 405, 488, 546, and 647 nm) and emission bandpass filters at 450/50, 515/30, 584/50, and 663/738 nm. For confocal microscopic study in situ, mice were injected intravenously with PE-conjugated anti-Ly6G/Ly6C at 0.2 mg/mL. The fluorescence intensity was monitored for 10 min under objective Plan Apo 20 × calibrated by each venule. Vessels selected for study were postcapillary venules. The total number of rolling leukocytes was quantified for each venule. A vertical line (100 μm) was drawn through each venule and all leukocytes crossing this line in 3 min was counted. Leukocyte rolling velocities were calculated by measuring the time necessary to travel a distance of 100 μm. Adherent cells were defined as leukocytes that did not move for at least 30s.

Rolling leukocytes was defined as those cells moving at a velocity less than that of the erythrocytes within a given vessel. The flux of rolling cells was determined as the number of rolling leukocytes passing a given point in the venule per minute. The rolling leukocyte results are expressed as cells/min. To evaluate leukocyte adhesion, leukocytes were considered adherent when they remained stationary for at least 30 s. The total leukocyte adhesion was quantified as the number of adherent cells observed within a 100-μm length of venule in a total time of 1 min and was expressed as cells/100 μm.

### Chemokines and cytokines measurement

Mice were pretreated with ENMD1068 (0.5 mg/kg, i.p.) 1 h before challenge with C-PAF (10^–7^ M/20 µL, i.n.). The supernatants obtained from BALF were collected at 4, 12, and 24 h. The levels of CXCL1 and CXCL2 was measured by ELISA using a commercially available ELISA DuoSet kit, in accordance with the manufacturer’s instructions. Sensitivity of the assays were 15.6 pg/mL (CXCL1), and 1.5 pg/mL (CXCL2). The cytokine levels are expressed as pg/mL.

### Co-immunoprecipitation and western blot

Protein interaction between PAR2 and PAFR was performed in RAW 264.7 cells. The cells were plated (1 × 10^6^ per well) 24 h before stimulation with PBS, C-PAF (100 nM), SLIGRL-NH_2_ (50 μΜ), or simultaneous stimulation with C-PAF (100 nM) + SLIGRL-NH_2_ (50 μΜ) mix for 20 min. After washing and resting, activated cells were lysed without agitation on ice for 30 min using RIPA buffer (in mM: 150 NaCl, 50 Tris–HCl, 5 EDTA, 2 Na and 1 MgCl_2_, pH 8.0; 1% Triton X-100, 1% NP-40, 1% sodium deoxycholate, 0.1% sodium dodecyl sulfate enriched with a protease inhibitor cocktail (SigmaFAST, Sigma-Aldrich, St. Louis, MO, USA; Cat# S8820). For the extraction of proteins, the cell lysates were centrifuged for 10 min at 14,000 *rpm* and 4 °C. The supernatant was collected and the protein concentration was measured by the Bradford assay. PureProteome™ Protein A/G Mix Magnetic Beads were used to obtain immunoprecipitates. Initially, 50 μL of magnetic spheres were incubated with anti-PAFR antibody for 3 h at 4 °C. After washing with 1 × PBS containing 0.1% Tween 20 (pH 7.4), the complex of beads and antibody was incubated with protein lysates (100 μg of protein per sample) overnight at 4 °C with constant agitation. As IgG control, protein extracts (100 μg) were also immunoprecipitated with normal rabbit IgG to assess the specificity of our antibodies. The precipitated proteins were eluted from the beads in elution buffer (0.2 M glycine–HCL, pH 2.5) and neutralized in neutralization buffer (1 M Tris–HCL, pH 8.5). The immunoprecipitated proteins were transferred to a 0.45 μm polyvinylidene fluoride (PVDF) Immobilon P membrane (MilliporeSigma, St. Louis, MO, USA; Cat# IPVH00010). The membrane was blocked at 37 °C with 5% bovine serum albumin (BSA) in PBS enriched with 0.1% Tween 20 before incubation with the primary anti-PAR2 antibody and anti-PAFR at 4 °C overnight. Secondary antibodies conjugated with peroxidase (Sigma-Aldrich, St. Louis, MO, USA) were added for 2 h. Immunodetection. was performed using enhanced chemiluminescence with the Luminatastrong™ Western HRP substrate (MilliporeSigma, St. Louis, MO, USA; Cat# WBLUF0100). Images were acquired using radiographic film or ChemiDoc XRS system (BioRad, RRID:SCR_019690) and quantification was performed using Image J software (NIH; RRID:SCR_003070).

### Analysis of intracellular calcium ion (Ca^2+^) mobilization

To analyze the effect of the PAR2 antagonist on intracellular Ca^2+^ mobilization induced by C-PAF, RAW 264.7 (1.5 × 10^5^ per well) murine macrophage cells were cultured in DMEM, supplemented with 10% fetal bovine serum and 1% penicillin/streptomycin at 37ºC in an atmosphere of 5% CO_2_. The cells were plated onto glass coverslips 24 h prior to imaging. These cells were incubated with ENMD-1068 (5 μM) or PBS for 30 min and loaded with 6 µM Fluo-4/AM for 15 min at 37 °C. Once transferred to a custom-built perfusion chamber on the stage of a Nikon C2 confocal microscope (Nikon, USA), the cells were perfused with HEPES^62^ buffer and stimulated with C-PAF (100 nM). Ca^2+^ signaling was monitored in these cells by excitation at 488 nm while collecting emitted light above 505 nm. Normalized amplitudes of the C-PAF-induced Ca^2+^ signals were extracted with ImageJ software (RRID:SCR_003070) and plotted as previously described^63^. Changes in fluorescence were normalized to baseline levels and used to analyze the amplitude of Ca^2+^ signals and the responding cells (n = 3 preparations with 30 cells per group).

### Confocal microscopy imaging

RAW 264.7 cells were plated (1.5 × 10^5^ per well) onto glass coverslips (22 × 22 mm) for 24 h before stimulation. The cells were pre-incubated with ENMD-1068 (5 μM) or PBS for 1 h and stimulated with C-PAF (100 nM) for 4 h. The glass coverslips were fixed with 4% paraformaldehyde for 20 min at room temperature, then permeabilized with 0.5% Triton buffer. Cells were incubated with blocking buffer (5% goat serum in 1% BSA/PBS) for 1 h and labeled with specific primary anti-PAR2 antibodies or anti-NF-kB overnight at 4ºC. Samples were subsequently incubated with species-specific secondary antibody conjugated to anti-rabbit IgG (568 nm) and anti-mouse (488 nm) (Invitrogen, Carlsbad, CA, USA). Nuclei were labeled with DAPI for 45 min at room temperature. The images were acquired by confocal microscopy at 63 × magnification using a model C2 microscope (Nikon, USA) and analyzed by Image J software (n = 3 preparations with 30 cells per group). Negative controls included cells incubated with normal anti-IgG.

### Quantitative real-time PCR

For analysis of mRNA expression, RNA was isolated from cells with TRIzol reagent (Ludwig Biotec, Alvorada, RS, Brazil). PAR2 expression (forward primer: 5′-ATGCGAAGTCTCAGCCTGG-3′) and (reverse primer: 5′-TGGGTTTCCAATCTGCCAATAAG-3′) expression was normalized to β-actin (forward primer: 5’-GGCTGTATTCCCCTCCATCG-3’) and (reverse primer: 5’-CCAGTTGGTAACAATGCCATGT-3’). The reverse transcription reaction was performed with 1 μg of total RNA using the iScript cDNA synthesis according to the manufacturer’s instructions (BioRad, Hercules, CA, USA; Cat# 1,708,890; RRID:SCR_008426) and relative messenger RNA (mRNA) expression of selected genes was quantified with and relative messenger RNA (mRNA) expression of PAR2 and β-actin (Integrated DNA Technologies, Coralville, Iowa, USA) genes was quantified in an ABI 7500 Real-time PCR (ThermoFisher, RRID:SCR_019334) by the comparative Ct method.

### Ethical standards

All the experimental procedures were approved by the UFMG Animal Ethics Committee (CEUA/UFMG 348/14) and are in accordance with ARRIVE guidelines.

### Supplementary Information

Supplementary Video 1.

Supplementary Video 2.

Supplementary Video 3.

Supplementary Legends.

Supplementary Information.

## Data Availability

All data that support the findings of this study are available from the corresponding author upon request, without privacy or ethical restrictions.
