# Supplementary figures and images for: Platelet-activating factor and protease-activated receptor 2 cooperate to promote neutrophil recruitment and lung inflammation through nuclear factor-kappa B transactivation
Source: Sci Rep. 2023 Dec 7;13:21637. doi: 10.1038/s41598-023-48365-1 (PMC10703791; doi:10.1038/s41598-023-48365-1)

## Slide 1
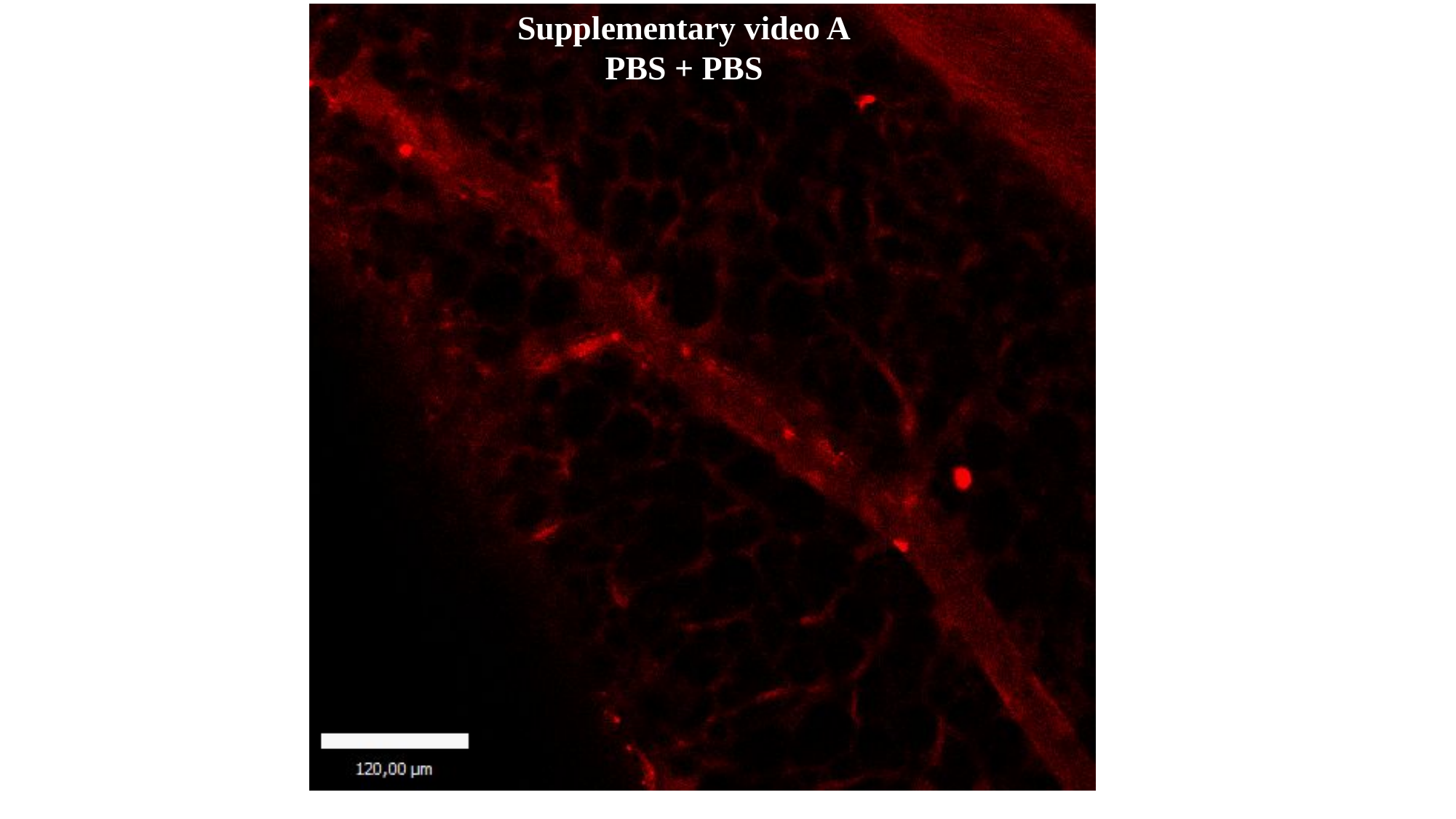

Supplementary video A
PBS + PBS

Supplement: Supplementary file 1 — Supplementary Video 1. [file 41598_2023_48365_MOESM1_ESM.pptx]

## Slide 1
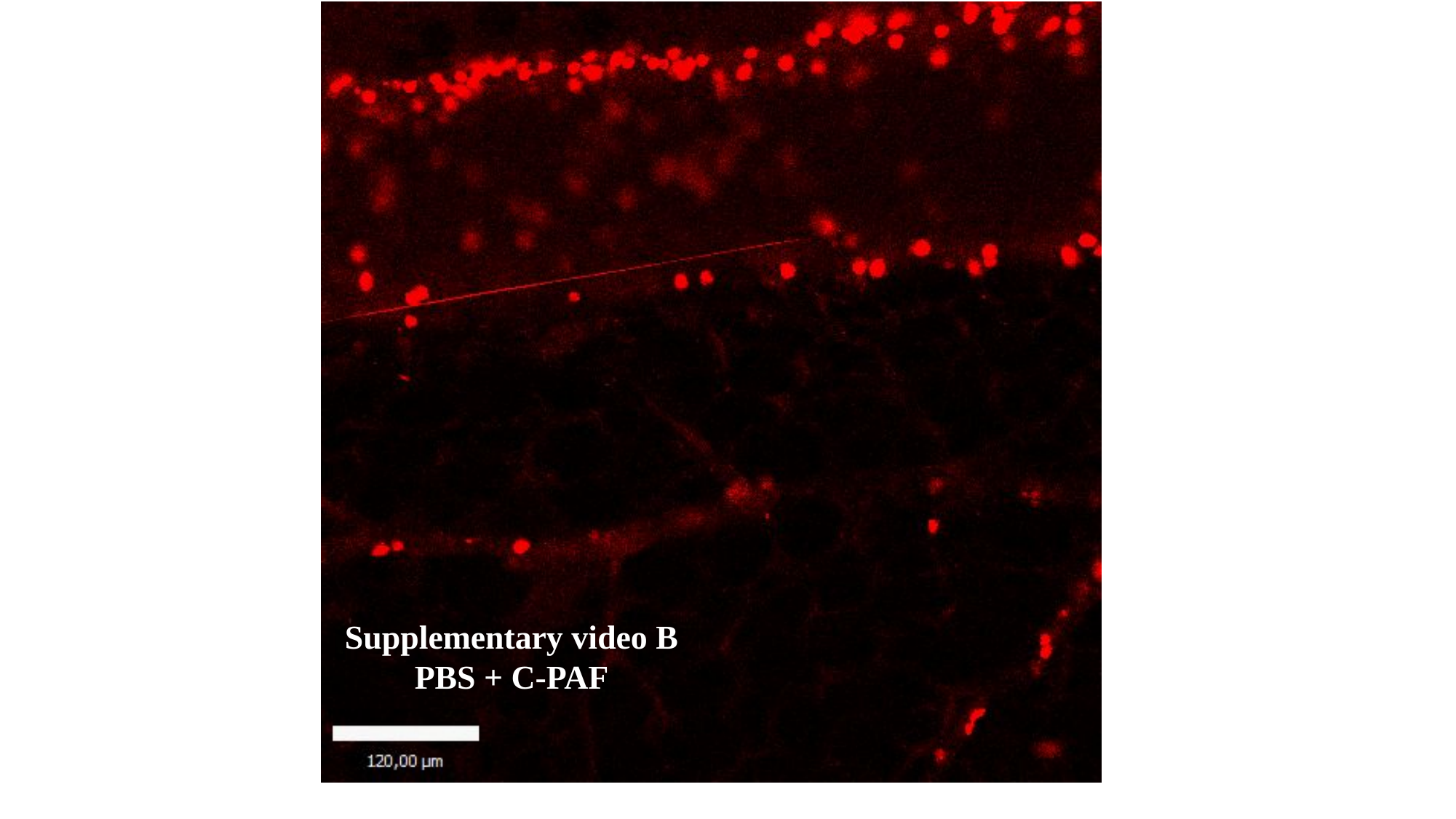

Supplementary video B
PBS + C-PAF

Supplement: Supplementary file 2 — Supplementary Video 2. [file 41598_2023_48365_MOESM2_ESM.pptx]

## Slide 1
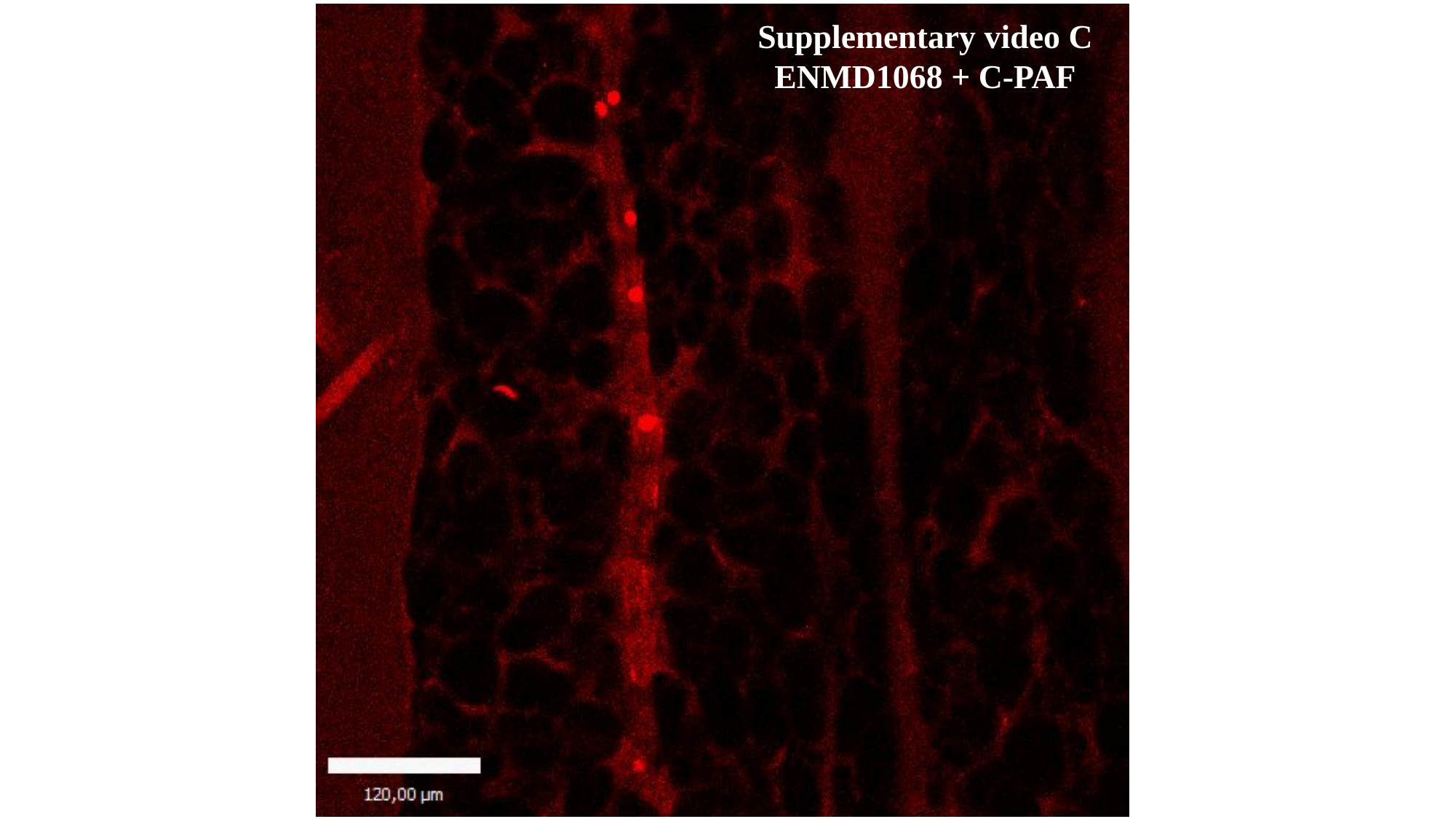

Supplementary video C
ENMD1068 + C-PAF

Supplement: Supplementary file 3 — Supplementary Video 3. [file 41598_2023_48365_MOESM3_ESM.pptx]
