# Supplementary material for: Platelet-activating factor and protease-activated receptor 2 cooperate to promote neutrophil recruitment and lung inflammation through nuclear factor-kappa B transactivation
Source: Sci Rep. 2023 Dec 7;13:21637. doi: 10.1038/s41598-023-48365-1 (PMC10703791; doi:10.1038/s41598-023-48365-1)
Supplement: Supplementary file 4 — Supplementary Legends. [file 41598_2023_48365_MOESM4_ESM.pdf]

Supplementary video legend

PAR2 blockade impairs rolling and adhesion of leukocytes in the mesentery microcirculation of mice. Representative video of mesentery microcirculation by intravital microscopy (neutrophils in red, anti-Ly6G/Ly6C-Gr-1; 0.2 mg/mL, i.v.), **A**: PBS group, **B**: C-PAF group and **C**: ENMD + CPAF group. Mice were pretreated with PBS ENMD1068 (0.5 mg/kg, i.p.) 1 h before stimulation with C-PAF (10^{-7} M, i.p.). After 4 h, rolling, velocity, and adhesion of leukocytes were evaluated in the venules of mesentery microcirculation. The fluorescence intensity was monitored for 10 min using an objective Plan Apo 20× calibrated by each venule. Scale bar: 120 μm.
