# Supplementary material for: Platelet-activating factor and protease-activated receptor 2 cooperate to promote neutrophil recruitment and lung inflammation through nuclear factor-kappa B transactivation
Source: Sci Rep. 2023 Dec 7;13:21637. doi: 10.1038/s41598-023-48365-1 (PMC10703791; doi:10.1038/s41598-023-48365-1)

SUPPORTING MATERIAL- CO-IMMUNOPRECIPITATION AND WESTERN BLOTTING

IP: PAFR e WB: PAR2

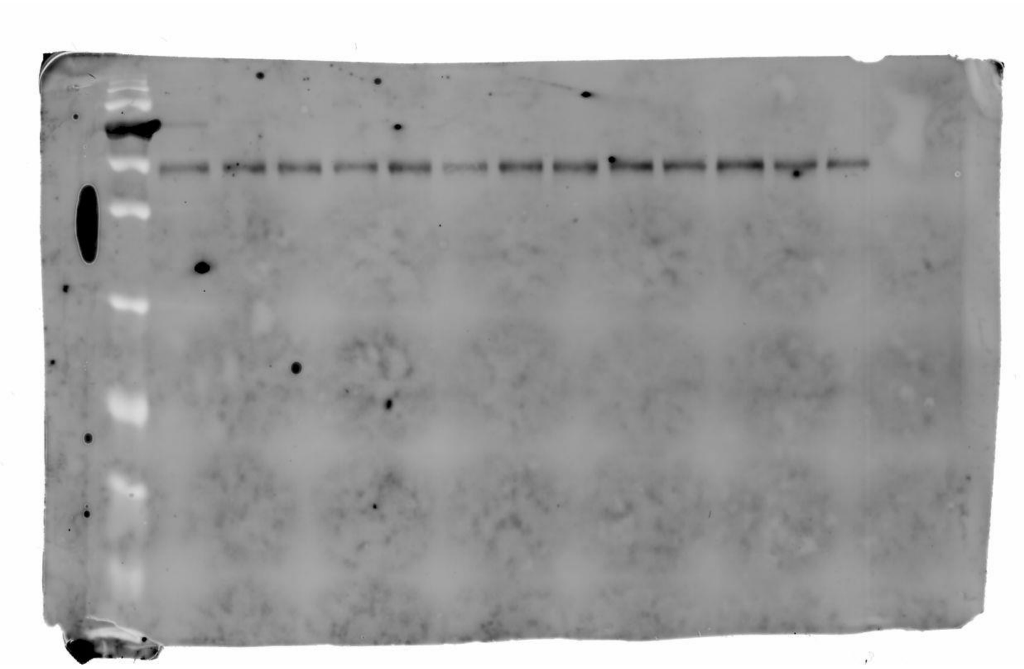

IP: PAFR e WB: PAFR

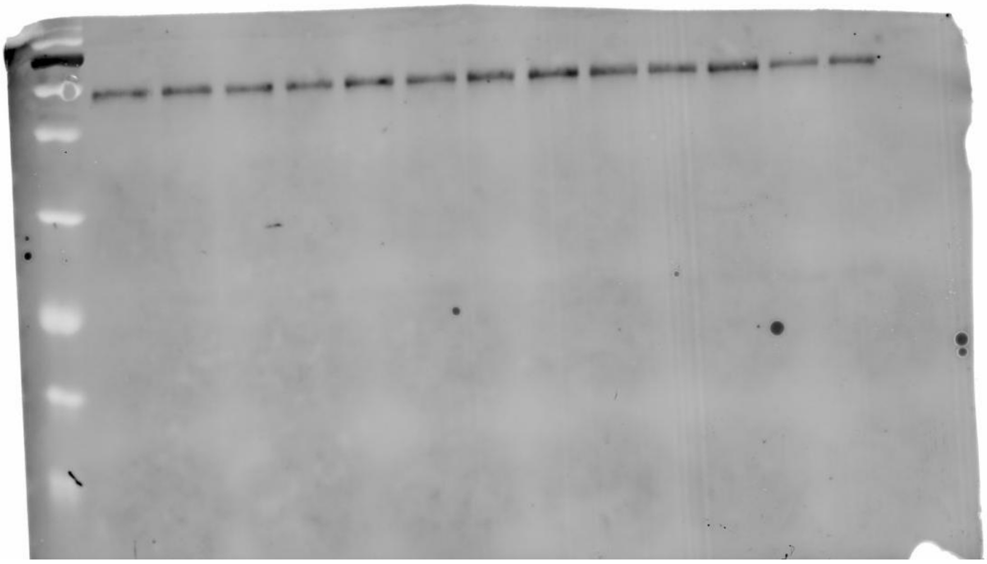

Supplement: Supplementary file 5 — Supplementary Information. [file 41598_2023_48365_MOESM5_ESM.pdf]
